# Transcriptome analysis of bacteriophage communities in periodontal health and disease

**DOI:** 10.1186/s12864-015-1781-0

**Published:** 2015-07-28

**Authors:** Tasha M. Santiago-Rodriguez, Mayuri Naidu, Shira R. Abeles, Tobias K. Boehm, Melissa Ly, David T. Pride

**Affiliations:** Department of Pathology, University of California, San Diego, 9500 Gilman Drive, MC 0612, La Jolla, CA 92093-0612 USA; Department of Medicine, University of California, San Diego, 9500 Gilman Drive, MC 0612, La Jolla, CA 92093-0612 USA; College of Dental Medicine, Western University of Health Sciences, Pomona, CA USA

**Keywords:** Saliva, Bacteriophage, Microbiome, Virome, Metagenome, Transcriptome, Periodontal Disease, Periodontitis

## Abstract

**Background:**

The role of viruses as members of the human microbiome has gained broader attention with the discovery that human body surfaces are inhabited by sizeable viral communities. The majority of the viruses identified in these communities have been bacteriophages that predate upon cellular microbiota rather than the human host. Phages have the capacity to lyse their hosts or provide them with selective advantages through lysogenic conversion, which could help determine the structure of co-existing bacterial communities. Because conditions such as periodontitis are associated with altered bacterial biota, phage mediated perturbations of bacterial communities have been hypothesized to play a role in promoting periodontal disease. Oral phage communities also differ significantly between periodontal health and disease, but the gene expression of oral phage communities has not been previously examined.

**Results:**

Here, we provide the first report of gene expression profiles from the oral bacteriophage community using RNA sequencing, and find that oral phages are more highly expressed in subjects with relative periodontal health. While lysins were highly expressed, the high proportion of integrases expressed suggests that prophages may account for a considerable proportion of oral phage gene expression. Many of the transcriptome reads matched phages found in the oral cavities of the subjects studied, indicating that phages may account for a substantial proportion of oral gene expression. Reads homologous to siphoviruses that infect Firmicutes were amongst the most prevalent transcriptome reads identified in both periodontal health and disease. Some genes from the phage lytic module were significantly more highly expressed in subjects with periodontal disease, suggesting that periodontitis may favor the expression of some lytic phages.

**Conclusions:**

As we explore the contributions of viruses to the human microbiome, the data presented here suggest varying expression of bacteriophage communities in oral health and disease.

**Electronic supplementary material:**

The online version of this article (doi:10.1186/s12864-015-1781-0) contains supplementary material, which is available to authorized users.

## Background

Periodontal disease is widespread and affects approximately half of the United States adult population [1]. It has been associated with systemic diseases such as atherosclerosis [2, 3], obesity [4], anemia of chronic disease [5], and diabetes [6–8], among other inflammatory conditions. The etiology of periodontal disease is likely multifactorial, with the oral microbiota playing a major role in its development. Many oral microbes have been linked to periodontitis, including *Porphyromonas gingivalis* [9], *Tannerella forsythia* [10], *Aggregatibacter actinomycetemcomitans* [11, 12], *Streptococcus mutans* [13], and *Treponema denticola* [14]. However, there are cases of periodontal disease in which none of these potential pathogens have been isolated from affected oral sites. The realization that there is a significant community of indigenous microbes inhabiting the human body, including many uncultivable bacteria [15–18], archaea [19], fungi [20], and viruses [21–24] has led some to hypothesize that periodontal disease may develop as a result of the oral microbial communities rather than the presence of any single pathogen [25–28].

Not only are bacterial communities in the human oral biofilm altered in periodontal disease [29, 30], but so too are oral viral communities [31]. Most studies of individual viruses associated with periodontal disease have been limited to cultivable eukaryotic viruses, such as Human Herpesvirus, Cytomegalovirus, and Epstein-Barr virus [32]. While studies have shown associations between herpesviruses and severe periodontal disease [33], a causal link has yet to be definitively demonstrated [34, 35]. We now know that human oral viruses are highly diverse, and most that have been identified are bacteriophages that predate upon bacteria and archaea instead of the human host [31, 36]. Phage communities are highly personalized features of individual human subjects and have distinct membership based on the oral biogeographic site from which they were derived [36, 37]. These communities of phages likely play an active role in shaping the ecology of oral cellular microbiota through lysis of their hosts and lysogenic conversions [21]. However, little is known about the transcriptional activity of these viral communities, or whether their patterns of expression reflect oral health status.

Despite the lack of studies characterizing oral phage gene expression, there have been efforts to characterize their bacterial counterparts [29, 38] and to associate patterns of transcription with disease severity [39]. Patterns of cellular microbial gene expression are altered when subjects with relative periodontal health are compared with those with significant periodontal disease. Among those genes upregulated in *Fusobacterium nucleatum*, *P. gingivalis*, and *A. actinomycetemcomitans* are those coding for chaperones, transposases and ABC transporters [38]. The abundance of some bacterial species is not associated with a similar increase in transcription from that species in the oral transcriptome, as was demonstrated for *Acinetobacter baumannii*; however, for other species such as *P. gingivalis*, *T. forsythia*, and *T. denticola*, relative abundance is associated with concomitant increases in transcription. These data suggest that there are factors other than oral health status that affect the transcription of the cellular microbiota, but it is unclear whether such a phenomenon exists for oral phage communities.

Although viruses make up a significant portion of the genes in any community, there are few studies focusing on the contribution viruses make to community gene expression. We sought to provide the first characterization of the contributions of viral communities to the oral transcriptome by comparing meta-transcriptomes with the virome sequences isolated from the same group of subjects. Our goals were to: 1) characterize viral transcription in the oral transcriptome, 2) identify specific viruses whose genes are transcribed in a cohort of human subjects, and 3) discern whether there are differences in viral gene expression patterns that correspond to oral health status.

## Results

### Human subjects and RNA enrichment

**Fig. 1:**
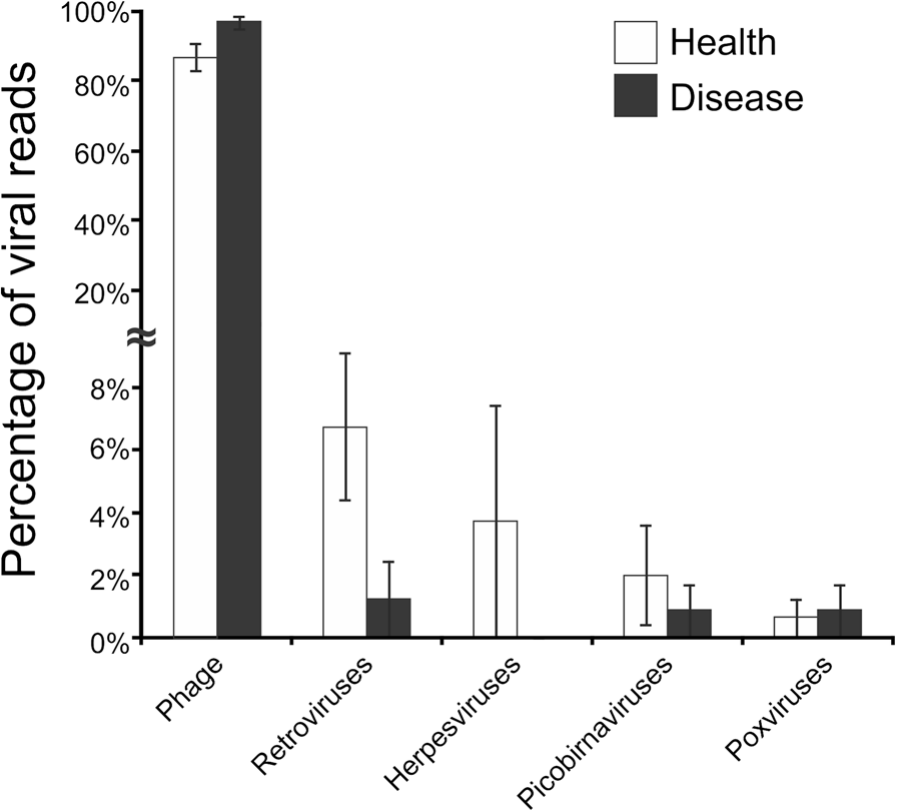
Bar chart representing the relative proportions (± standard error) of viral reads assigned to phages, retroviruses, herpesviruses, picobirnaviruses, and poxviruses. The y-axis represents the percentage of viral reads assigned to each category

We recruited 16 human subjects and sampled their saliva. Nine of the subjects had relative periodontal health and the other 7 had significant periodontal disease (Additional file 1: Table S1). We previously utilized this same cohort of individuals to characterize the virome of the oral biofilm in subjects with oral health and periodontal disease [31]. We isolated and enriched total RNA from each subject to remove most human and ribosomal RNAs. We then sequenced a total of 24,004,870 reads (mean 1,500,304 per subject) of average length 96 nucleotides (Additional file 1: Table S2); of the 24 million reads, 10,814,865 (45.10 %) had identifiable homologues. The vast majority of the transcriptome reads were homologous to sequences associated with bacteria (10,122,534), while only 654,399 (6.05 %) were homologous to sequences associated with eukaryote DNA, indicating the success of the enrichment process. There were 20,333 (0.19 %) that were homologous to sequences associated with archaea and 17,599 (0.16 %) homologous to sequences associated with viruses (mean of 1099 reads per subject). Some eukaryotic virus gene expression was identified including sequences associated with virus families Herpesviridae, Retroviridae, Poxviridae, and Picobirnaviridae (Fig. 1), but the vast majority of virus gene expression was associated with bacteriophages. There were significantly (*p* = 0.02) more sequence reads homologous to phages in subjects with relative periodontal health than those with significant periodontal disease (Additional file 2: Figure S1). Phage genes involved in replication, virulence, lysis, and lysogeny had more homologues in periodontally healthy subjects (Additional file 2: Figure S2), suggesting that oral health may be the preferred state for the transcription of oral phages.

### Taxonomic distributions found in oral phage RNA

We characterized the transcriptome reads homologous to Caudovirus families to determine if the expression of certain bacteriophages may be favored in oral health or periodontal disease. The vast majority were homologous to siphoviruses (63.8 % in health and 72.9 % in disease), many of which integrate into bacterial genomes (Table 1) [40]. A relative minority of the phages found were homologous to myoviruses (3.0 % in health vs 4.1 % in disease) and podoviruses (12.1 % in health vs 9.5 % in disease). Those that could not be classified into Caudovirus families represented 7.4 % of the viruses in health and 10.6 % in disease. None of the differences in phage families identified between oral health and periodontal disease were statistically significant.

**Table 1:** Percentage of reads assigned to phage families

	Health	Disease
Siphoviridae	63.8 %	72.9 %
Myoviridae	3.0 %	4.1 %
Podoviridae	12.1 %	9.5 %
Unclassified	7.4 %	10.6 %

**Fig. 2:**
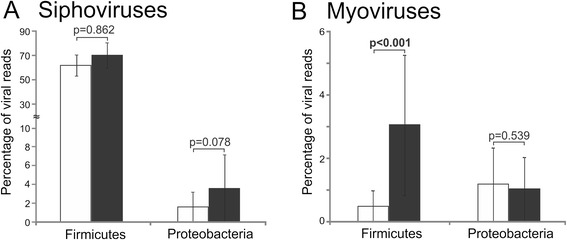
Bar graph of the percentage of phage reads (± standard error) homologous to Firmicutes or Proteobacteria from the phage family Siphoviridae (**a**) or Myoviridae (**b**). Subjects with relative periodontal health are shown in white bars and subjects with significant periodontal disease are shown in black bars. *P*-values are demonstrated above each bar and represent the observed differences between periodontal health and disease, and significance (*p* < 0.05) is shown in bold

We profiled the transcriptomes by analyzing the bacterial host taxonomy at the phylum level of BLASTX homologues to each phage. Transcriptome reads homologous to phages from Firmicutes and Proteobacteria were amongst the most highly expressed in health and disease. We focused on Caudovirus families that were highly expressed in our subjects to determine whether certain families might have greater expression based on their BLASTX profiles. Among the siphoviruses, there was no significant difference in those expressed that were homologous to Firmicute viruses (61.9 % in oral health *vs.* 70.2 % in periodontal disease; *p* = 0.86), although there was a non-significant trend found for Proteobacteria (1.6 % in oral health *vs.* 3.6 % in periodontal disease; *p* = 0.08) (Fig. 2a). Among the myoviruses, there were no significant differences found in those homologous to Proteobacteria viruses (1.2 % in oral health *vs.* 1.0 % in periodontal disease; *p* = 0.54), but there were substantial differences found in the Firmicutes (0.5 % in oral health *vs.* 3.0 % in periodontal disease; *p* < 0.001) (Fig. 2b). While there are exceptions, myoviruses often are lytic, and the higher expression of putative myoviruses from Firmicutes in subjects with periodontal disease suggests that they may play a role in shaping Firmicute ecology [41]. Interestingly, a previous study of oral viromes of the same subjects showed a predominance of putative myoviruses in the subgingival crevice in subjects with significant periodontal disease [31].

### Expression of individual viruses in the oral cavity

**Fig. 3:**
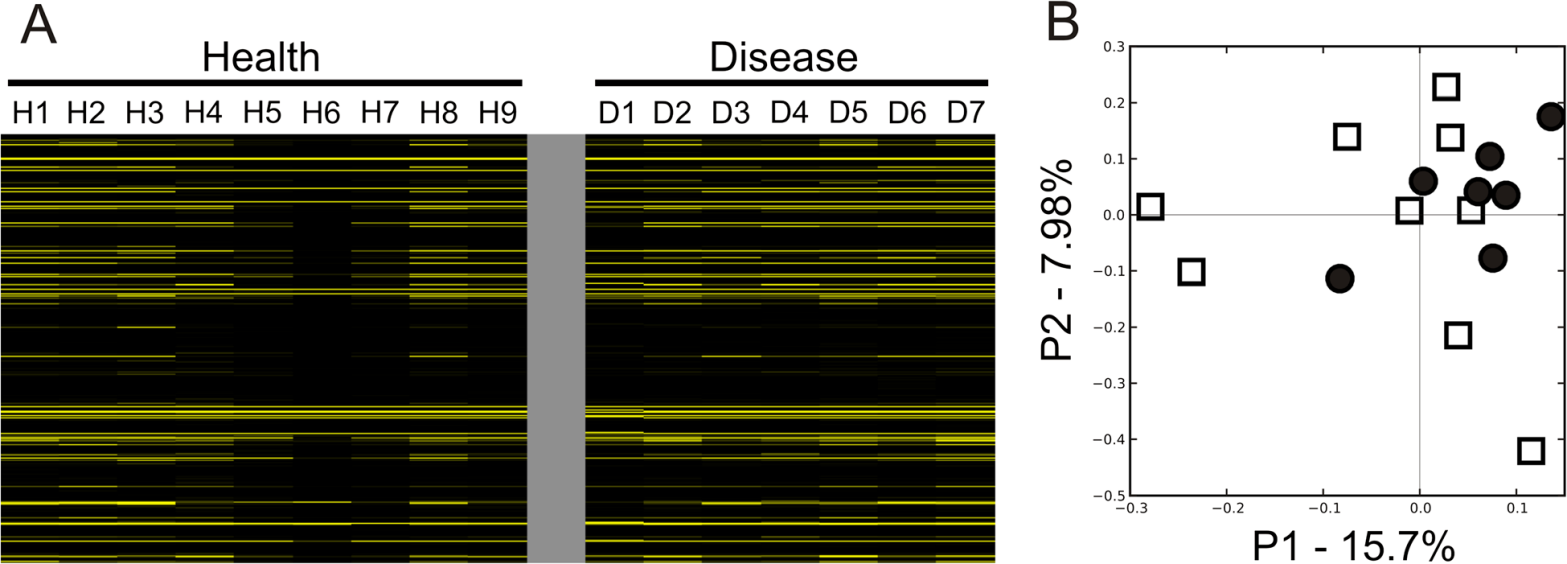
Heatmap (**a**) and principal coordinates analysis (**b**) of transcriptome reads matching viral contigs from all subjects. The heatmap is organized by subject and oral health status, where each column represents the individual subjects. Each row represents a unique phage contig. For the principal coordinates analysis, white boxes represent subjects with relative periodontal health and black circles represent subjects with significant periodontal disease

**Fig. 4:**
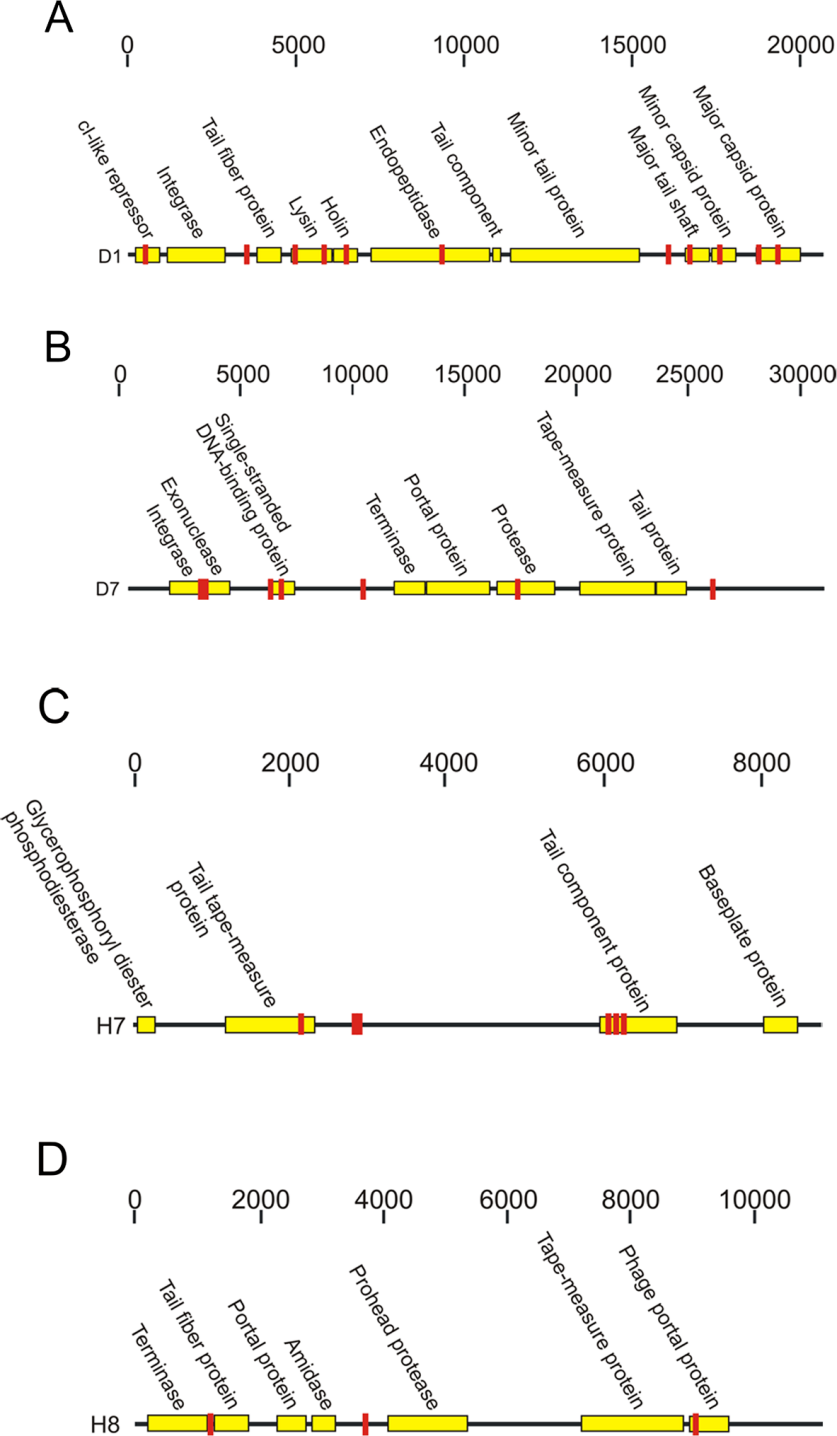
Diagram of bacteriophage genomes that were expressed from subjects #D1 (**a**), #D7 (**b**), #H7 (**c**), and #H8 (**d**). Putative ORFs are indicated by the yellow boxes and their putative functions based on BLASTX homology (E-score < 10^−5^) shown above each box. The length of each contig is denoted at the top of each panel, and areas to which transcriptome reads matched each virus are demonstrated in red

Because only a minority of the total gene expression in our subjects was homologous to known phages, we were unable to assemble viruses to determine which were expressed. However, we previously sequenced the viromes from these same subjects [31], and were able to compare the transcriptomes to these viruses to determine which were expressed. The percentage of transcriptome reads that matched viruses from these subjects far exceeded those that had homologues in the SEED database (Additional file 2: Figure S3). Approximately 30 % of the transcriptome reads from healthy subjects and 26 % of the transcriptome reads from subjects with periodontal disease matched viruses from those same subjects, indicating that the expression of oral phages likely far exceeded that which could be detected based on homology to known phages. Many of the transcripts mapped to the exact same viral contigs, suggesting that some similar phages were present and expressed in all subjects (Fig. 3a). As observed by principal coordinates analysis, there was less variation observed in phage gene expression among the subjects with periodontal disease, but this variation was not distinct from that observed in periodontal health (Fig. 3b). Many of the viral contigs that matched transcriptome reads had no or only a single identifiable homologue to other known phages. There were, however, several phages that had numerous identifiable homologues across their genomes and had transcripts that matched their genome sequences (Fig. 4). For example in periodontal disease, the capsid, peptidase, holin, lysin, and tail fibers from a 22 kb phage were expressed in subject #D1 (Fig. 4a). We also identified similar expression across the genome of a 32 kb phage in subject #D7, where a protease, single-stranded DNA binding protein, exonuclease, and integrase were expressed (Fig. 4b), suggesting their probable ability to integrate into the host genome. We also found phages with expression in healthy subjects #H7 (Panel c) and #H8 (Panel d). These data verify that individual phages from the oral cavities of our subject groups were expressed in periodontal health and disease.

### Gene expression in lytic and lysogenic cycles

**Fig. 5:**
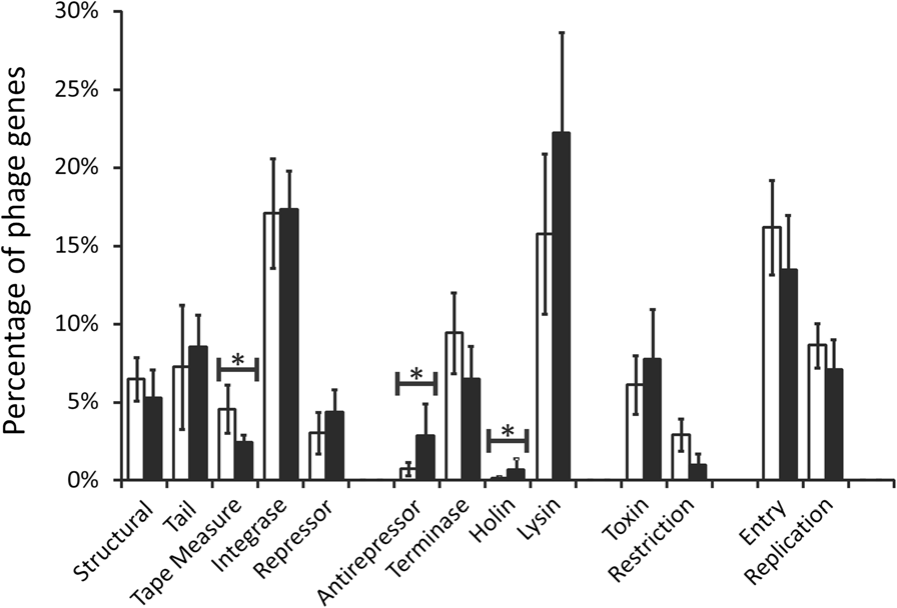
Bar plots (± standard deviation) of phage genes expressed in relative periodontal health (white bars) or significant periodontal disease (black bars). Each functional category of genes is indicated on the x-axis and the percentage of total viral genes expressed is demonstrated on the y-axis. *P*-values that are  < 0.05 are represented by the '*' and represent comparisons between subjects with periodontal health and disease

We investigated the gene expression profiles of phages in our subject groups to determine whether there may be differences in gene expression in periodontal health and disease. By focusing on those gene functions generally associated with lysogenic lifestyles (*i.e.* integrases/transposases, and repressors), we compared gene expression by oral health status. We found that there were few significant differences in each of these gene categories by health status, with only tape measures (*p* = 0.003) more highly expressed in subjects with relative periodontal health (Fig. 5). We also investigated the relative expression of viral genes associated with the lytic cycle in most phages. We found that antirepressors (*p* < 0.001) and holins (*p* < 0.001) were significantly more highly expressed in subjects with periodontal disease. While lysins were not significantly more highly expressed, there was a trend towards greater expression in subjects with periodontal disease (15.8 % in periodontal health *versus* 22.2 % in periodontal disease).

## Discussion

The study of human viral communities is in its relative infancy compared to human cellular microbiota. The majority of studies characterize bacterial communities by 16S rRNA amplicon sequencing, while far fewer utilize metagenome or transcriptome sequencing. In contrast to microarrays, RNA sequencing does not require pre-existing genome sequence data from each microbe present, but rather can characterize gene expression based on sequence homology to existing databases. The data presented here provide a first glimpse into the *in vivo* behavior of the diverse communities of phages that inhabit the human oral cavity. However, despite the benefits, its main limitation is in a relative inability to discern phage sequences from the abundant bacterial and archaeal sequences present. Our data illustrate this point, where only a minority of the sequence reads have homology to known viruses, including phages in the SEED database compared to bacteria (Additional file 1: Table S2), while a far greater proportion of the reads match viruses assembled from the viromes of each subject (Additional file 2: Figure S3). We identified only 0.16 % of the reads homologous to known viruses in this study, which is greater than a recent oral microbiome study where only 0.003 % of their reads were homologous to known viruses [25]. That study, however, did not compare their metagenome reads to viromes from the same subjects, which likely would have demonstrated that a far greater proportion of their metagenomes were attributable to viruses. Viral communities are known in a number of different environments to harbor gene functions also present in their bacterial hosts [21, 42], which could result in an overestimation of the gene functions we ascribed to phages in this study. Conversely, when these genes are not properly annotated as phages in their host bacterial genomes, they could result in an underestimation of the proportion of the transcriptome sequences attributable to phages.

There are significant differences between viral communities in periodontal health and disease [37], but prior studies have had limited insight into the gene expression of viruses in the oral cavity. The data produced in this study suggest that some lytic modules are active in subjects with significant periodontal disease (Fig. 5). Lysins were among the most highly expressed phage genes, and they were differentially expressed in periodontal health and disease, although the results were not statistically significant. We hypothesize from these data that the inflammatory environment in periodontal disease may support the lytic cycle of some temperate phages, which may be related to bacterial stress. Shifts in pH, the presence of heavy metals and toxic compounds are known to cause bacterial stress and consequently the production of virus-like particles in certain bacteria [43]. Periodontal disease is also known to promote bacterial stress, resulting in bacterial resistance to acidic or alkaline conditions; yet, how this potentially triggers the lytic cycle of temperate phages remains to be addressed [44]. While a link between oral health status and viral lytic/lysogenic functions needs to be demonstrated experimentally, the data from this study provides an intriguing basis to support further study. A similar study with the same group of subjects showed a high proportion of putative myoviruses in the subgingival crevices of subjects with periodontal disease [31], suggesting that the lytic cycle may be preferred in subjects with significant periodontal disease. Perhaps the significant proportion of lysins identified in our cohort suggests a “kill the winner” dynamic in subjects with significant periodontal disease, but a longitudinal study with a larger group of subjects would be necessary to support that hypothesis.

While this study provides evidence for differential gene phage expression in subjects with periodontal health and disease, the are some confounders in the subject population that could affect the results. The age of the subjects studied was significantly different, where subjects with periodontal health had a mean age of 35 years, and subjects with periodontal disease had a mean age of 60. Very little is known about the effects of age on the human oral virome; however, in a prior study of subjects with periodontal health, we observed no impacts of age on the oral virome [45]. In fact, we found that there were significant numbers of shared phage between household members regardless of their ages. Similarly, we found no specific effect of ethnicity on virome contents, but none of our virome studies [21, 31, 36, 37, 45, 46] have been powered adequately to characterize any potential effects of ethnicity on the human oral virome. In this study, most of the subjects with periodontal disease were Caucasian compared to a number of different ethnicities amongst those subjects with periodontal health; these subject characteristics largely reflect the patient population at our study site. A much larger study focused on the effects of age and ethnicity is needed to characterize any effects of potential confounders on the study of the human oral virome.

This study offers some benefits not found in other RNA-sequencing studies, including that we sequenced the RNA from a diverse group of different subjects and demonstrated trends across subject groups, and we enriched the RNA to remove many ribosomal and human RNAs to characterize a more robust dataset. Because the phages characterized across each subject or subject group were highly diverse, traditional techniques such as RT-PCR to verify trends seen in sequence data were not possible. For example, all subjects have integrases, lysins, repressors, and tail fibers, but most of these gene sequences are unique to each subject and phage; therefore, gene expression could only be reproduced from a single phage using RT-PCR, which would not capture trends seen throughout that community. Thus, while the expression of any individual phage may vary, the overall expression in the community may show significant trends.

There were some gene expression differences across the phage communities in periodontal health and disease (Fig. 5), despite there being roughly similar proportions of phage families in each state (Table 1). Genes associated with penetration of the cell membrane (holins), promotion of lytic gene expression (antirepressors), and lysis (lysins) were expressed at higher levels in periodontal disease than in periodontal health (Fig. 5). This may support a preference of certain phages for the lytic cycle in periodontal disease, although experimental demonstration would be necessary to prove such a phenomenon. Most genes associated with lysogeny, including integrases and repressors, were not significantly different based on oral health status. Notably, there was no concerted expression of phage holins and lysins in periodontal health or disease. Some phages lyse their hosts using a holin-endolysin system; however, in others, there is no interaction between the holin and the endolysin genes in host lysis [47]. Some of these endolysins are secreted to the extracellular environment before completion of the viral lysis cycle independent of holin activity. Additionally, some holin genes are not located downstream of lysins [48], suggesting that holin and lysin gene expression in these phages is independent.

We believed that by analyzing the viromes in each of these subjects, we would find that the most abundant viruses in each subject also would have the greatest gene expression. Indeed, a relative minority of the phages present in each subject had detectable transcripts. Many of the transcriptome reads from different subjects with periodontal health and disease matched the same viruses, suggesting that there is some conservation of phage genes across all subjects. This phenomenon has previously been demonstrated in separate cohorts in the saliva of healthy human subjects and in the dental plaque of subjects with periodontal health and disease [31, 37]. We believe that part of the explanation why only a minority of the phages in each cohort had identifiable transcripts is related to the relative longevity of viruses in the oral cavity. We recently demonstrated that human oral viruses are remarkably persistent over short and long time periods [37, 49], but transcription from these viruses was not necessarily detected. The longevity of these viruses may not be directly related to their ongoing transcription and production of virions, but rather their ability to attach and persist on mucosal surfaces [49]. Thus, the persistence of oral viruses may be secondary to their ability to attach to surfaces, which would reduce their need for ongoing replication and transcription to produce virions.

## Conclusions

We know very little about the role of viruses on human body surfaces, but the oral virome now is coming into greater context. The human oral cavity is populated by numerous bacteriophages that carry substantial gene functions putatively involved in the pathogenesis of their cellular hosts [21, 22]. Many phages are highly persistent members of the oral microbiome, with transience the exception rather than the rule [37]. Oral phages are also highly specific to individual, biogeographic site, host-sex, and oral health status [31, 37]. The RNA-sequencing data here indicates that there is substantial transcriptional activity from oral phage communities, and that oral health status may play some role in the activity of viruses. While there does not appear to be great differences in the expression of the lysogenic modules of oral phages, lytic module expression is somewhat increased in subjects with significant periodontal disease. The development of a model oral environment to investigate the interactions between phages and their cellular hosts would be ideal to substantiate the role and activity of the oral phage community in periodontal health and disease.

## Methods

### Ethics statement

Subject recruitment and enrollment was approved by the University of California, San Diego and the Western University Administrative Panels on Human Subjects in Medical Research. All subjects signed informed consent indicating their willingness to participate in this study.

### Study criteria and sample collection

Each subject underwent a baseline periodontal examination including measurements of probing depths, clinical attachment loss, Gingival Index, Plaque Index, and gingival irritation [50], and their oral health status was recorded (Additional file 1: Table S1). We used the 1999 International Workshop for Classification of Periodontal Diseases and Conditions, where periodontitis including juvenile forms of periodontitis is defined by loss of attachment. For a diagnosis of healthy, all sites had to have an attachment level of 0 mm and an absence of bleeding on probing. We excluded attachment levels from sites that were located next to 3^rd^ molars, edentulous areas and sites where attachment loss was clearly caused by factors other than periodontal disease such as chronic toothbrush trauma. For those subjects in this study diagnosed as having moderate gingivitis and mild periodontitis, they met criteria for periodontal health according to this classification scheme. Approximately 3 ml of saliva was collected from each subject without stimulation, immediately frozen on dry ice and stored at −20 °C until use in this study. All subjects completed a questionnaire detailing their dietary habits and other co-morbidities. Exclusion criteria included preexisting medical conditions that could result in substantial immunosuppression.

### RNA extraction, enrichment and sequencing

Total RNA was extracted from each subject using the Mirvana kit (Life Technologies, Grand Island, NY), with the inclusion of a bead-beating step for 20 min with Lysing-Matrix B (MP Bio, Santa Ana, CA). Total RNA then was enriched for microbial RNA using MicrobEnrich (Life Technologies), and further enriched for mRNA using MicrobExpress (Life Technologies) and MegaClear (Life Technologies), which are designed to remove ribosomal RNAs. Enriched RNA then was prepared for sequencing through the construction of cDNA libraries using the Ion Total RNA-Seq kit (Life Technologies), and subjected to successive rounds of Ampure bead purification (Beckman-Coulter, Brea, CA) to remove small cDNAs. Libraries were quantified using an Agilent Bioanalyzer HS DNA Kit (Agilent, Santa Clara, CA) and then were sequenced on 316 chips using an Ion Torrent Personal Genome Machine [51], producing an average of 1,500,304 reads per subject of mean length 96 nucleotides. All sequences are available for download in the MG-RAST database (metagenomics.anl.gov/) under the project #5691 or under the name ‘Health_And_Disease_Transcriptome_Project.’

### Processing of RNA sequences

Sequencing reads were trimmed according to modified Phred quality scores of 0.5 using CLC Genomics Workbench 6.51 (CLC bio USA, Cambridge, MA). The remaining reads were further processed for quality control by removing reads with substantial length variation (reads <50 nucleotides or >200 nucleotides), or reads where ≥25 % of the length was due to homopolymers tracts. Each transcriptome was screened for contaminating human nucleic acids using BLASTN analysis (E-score <10^−5^) against the human reference database available at ftp://ftp.ncbi.nlm.nih.gov/genomes/H_sapiens/. Any reads homologous to human sequences were removed prior to further analysis.

### Analysis of transcriptomes

Analysis of gene expression was determined by BLASTX analysis of the SEED database using MG-RAST (E-score <10^−5^) [52]. Statistical significance was determined by comparing the means for all subjects for all subsystems between subject groups by two-tailed t-tests using Microsoft Excel 2007 (Microsoft Corp., Redman, WA). Analysis of the expression differences between subject groups for individual genes also was determined by comparisons of means. The data for each individual gene were compared between MG-RAST and the normalized RPKM values obtained from CLC Genomics Workbench 6.51 to verify that they produced similar results. The relative abundances of virus families were determined by BLASTX analysis of the SEED database using MG-RAST [52]. Phages were classified into Caudovirus families *Myoviridae*, *Siphoviridae*, and *Podoviridae* based on homology to known viruses from these families as previously described [53]. Analysis of transcription of individual viruses was determined by mapping transcriptome reads against a database of virome contigs from each subject [31]. Read mappings were performed at 98 % identity over the entire length of each sequenced read, which were defined as matches. Heatmaps were generated based on the normalized proportions of reads mapping to pooled viromes from each subject, and were visualized using JAVA Treeview [54]. Principal coordinates analysis was performed using binary Sorensen distances using Qiime 1.1 [55]. Statistical comparisons of means were performed by two-tail t-tests and equality of variances for data that was not normally distributed was performed by F-tests. Each was determined using Microsoft Excel 2007 (Microsoft Corp., Redman, WA).

## Additional files

Additional file 1: Table S1. Study subjects. **Table S2.** Transcriptome reads from all subjects. (PDF 39 kb) Additional file 2: Figure S1. Bar plots of the total number of identifiable phage genes expressed in relative periodontal health or significant periodontal disease. **Figure S2.** Number of transcriptome reads with identifiable phage homologues in subjects with periodontal health (white bar) or disease (black bar). **Figure S3.** Percentage (±standard deviation) of transcriptome reads from subjects with periodontal health (white bar) or disease (black bar) that were homologous to virome sequences from all subjects. (PDF 365 kb)
