# Supplementary material for: Transcriptome analysis of bacteriophage communities in periodontal health and disease
Source: BMC Genomics. 2015 Jul 28;16(1):549. doi: 10.1186/s12864-015-1781-0 (PMC4515923; doi:10.1186/s12864-015-1781-0)
Supplement: Additional file 1: Table S1. — Study subjects. Table S2. Transcriptome reads from all subjects. (PDF 39 kb) [file 12864_2015_1781_MOESM1_ESM.pdf]

Supplemental Table 1. Study subjects.

Subject	Age	Ethnicity	Sex	Diagnosis
H1	25	Asian	M	Health
H2	25	Caucasian	M	Health
H3	51	Latino	F	Moderate Gingivitis
H4	34	African American	M	Health
H5	32	Asian	F	Health
H6	24	Asian	F	Health
H7	27	Caucasian	M	Health
H8	44	Caucasian	F	Chronic Mild Generalized Periodontitis
H9	50	Latino	F	Chronic Mild Generalized Periodontitis
D1	73	Caucasian	M	Chronic Severe Generalized Periodontitis
D2	66	Caucasian	M	Chronic Severe Generalized Periodontitis
D3	62	Caucasian	F	Chronic Moderate Generalized Periodontitis
D4	69	Caucasian	M	Chronic Severe Generalized Periodontitis
D5	48	Caucasian	F	Chronic Moderate Generalized Periodontitis
D6	73	Caucasian	M	Chronic Severe Generalized Periodontitis
D7	27	Asian	M	Generalized Aggressive Mild Periodontitis

Supplemental Table 2. Number of transcriptome reads with homologues in the SEED database

Subject	Archaea	Bacteria	Eukaryota	Viruses	Other
Health					
H1	0.17% (1304)	93.71% (719052)	4.94% (37876)	0.10% (780)	1.08% (8271)
H2	0.13% (1162)	96.02% (831492)	2.47% (21395)	0.07% (565)	1.31% (11326)
H3	0.28% (1439)	94.93% (483677)	3.71% (18922)	0.09% (457)	0.98% (5008)
H4	0.16% (1376)	88.68% (767227)	9.66% (83569)	0.14% (1224)	1.36% (11751)
H5	0.21% (1964)	90.45% (838305)	8.81% (81620)	0.09% (856)	0.44% (4032)
H6	0.22% (1431)	78.80% (503607)	16.34% (104442)	0.28% (1777)	4.35% (27830)
H7	0.17% (790)	89.41% (406158)	3.17% (14418)	0.20% (890)	7.04% (32002)
H8	0.16% (1032)	81.11% (517909)	15.96% (101939)	0.13% (826)	2.64% (16834)
H9	0.22% (1411)	95.9% (608531)	3.43% (21790)	0.38% (2441)	0.06% (386)
Disease					
D1	0.01% (36)	95.99% (338077)	3.80% (11196)	0.072% (246)	0.76% (2660)
D2	0.28% (1749)	93.83% (595916)	3.81% (24195)	0.14% (918)	1.94% (12347)
D3	0.17% (1324)	93.81% (715349)	3.00% (22903)	0.23% (1767)	2.78% (21199)
D4	0.16% (899)	94.22% (541845)	3.24% (18657)	0.24% (1345)	2.15% (12340)
D5	0.19% (830)	95.25% (409507)	2.74% (11765)	0.19% (822)	1.63% (6992)
D6	0.17% (1824)	95.41% (1030009)	2.78% (30042)	0.20% (2174)	1.44% (15589)
D7	0.20% (1762)	93.23% (815873)	5.68% (49670)	0.06% (511)	0.84% (7326)
