# Supplementary material for: Transcriptome analysis of bacteriophage communities in periodontal health and disease
Source: BMC Genomics. 2015 Jul 28;16(1):549. doi: 10.1186/s12864-015-1781-0 (PMC4515923; doi:10.1186/s12864-015-1781-0)
Supplement: Additional file 2: Figure S1. — Bar plots of the total number of identifiable phage genes expressed in relative periodontal health or significant periodontal disease. Figure S2. Number of transcriptome reads with identifiable phage homologues in subjects with periodontal health (white bar) or disease (black bar). Figure S3. Percentage (±standard deviation) of transcriptome reads from subjects with periodontal health (white bar) or disease (black bar) that were homologous to virome sequences from all subjects. (PDF 365 kb) [file 12864_2015_1781_MOESM2_ESM.pdf]

Supplemental Figure 1

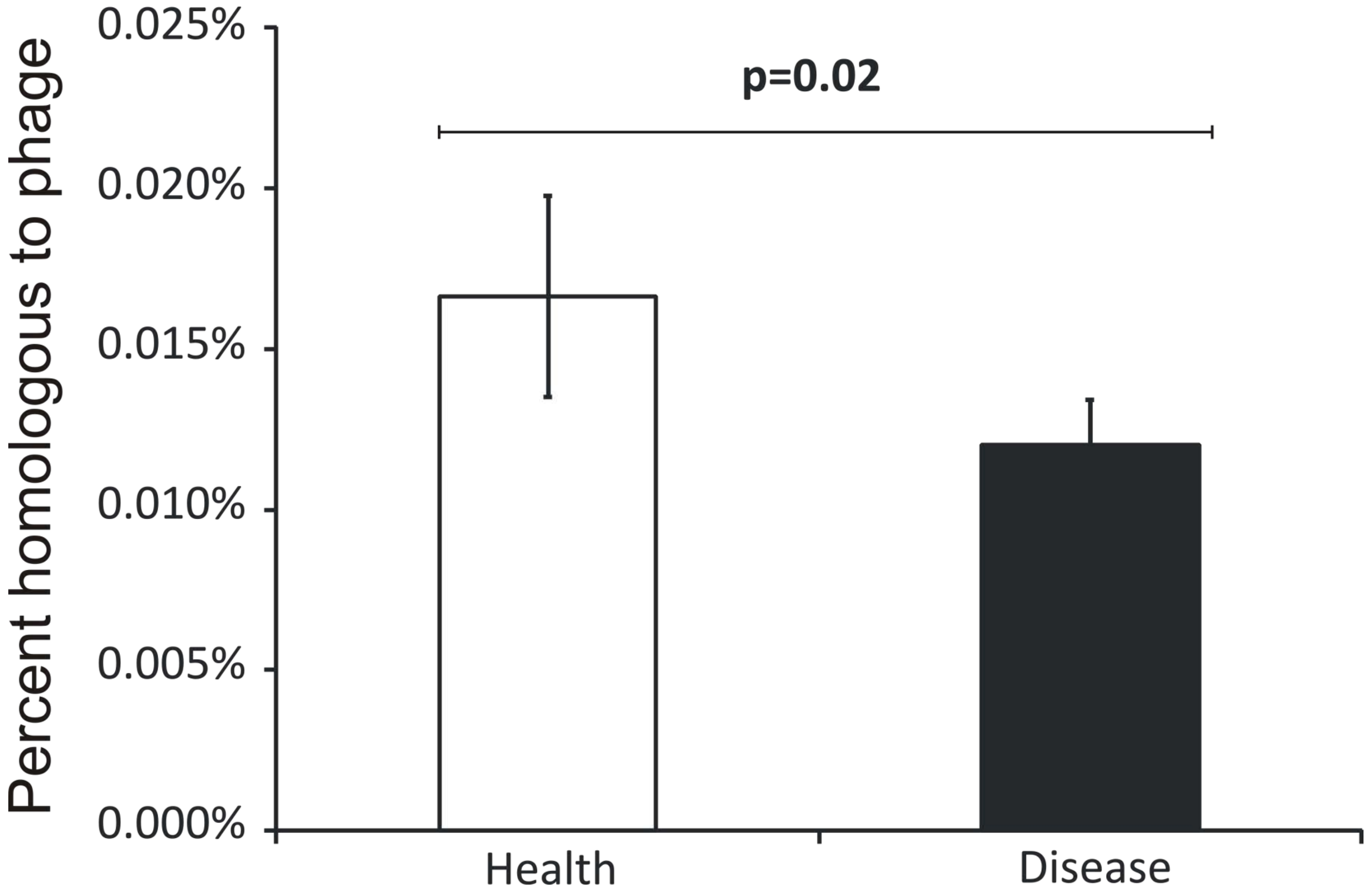

Supplemental Figure 1: Bar plots of the total number of identifiable phage genes expressed in relative periodontal health or significant periodontal disease.

Supplemental Figure 2

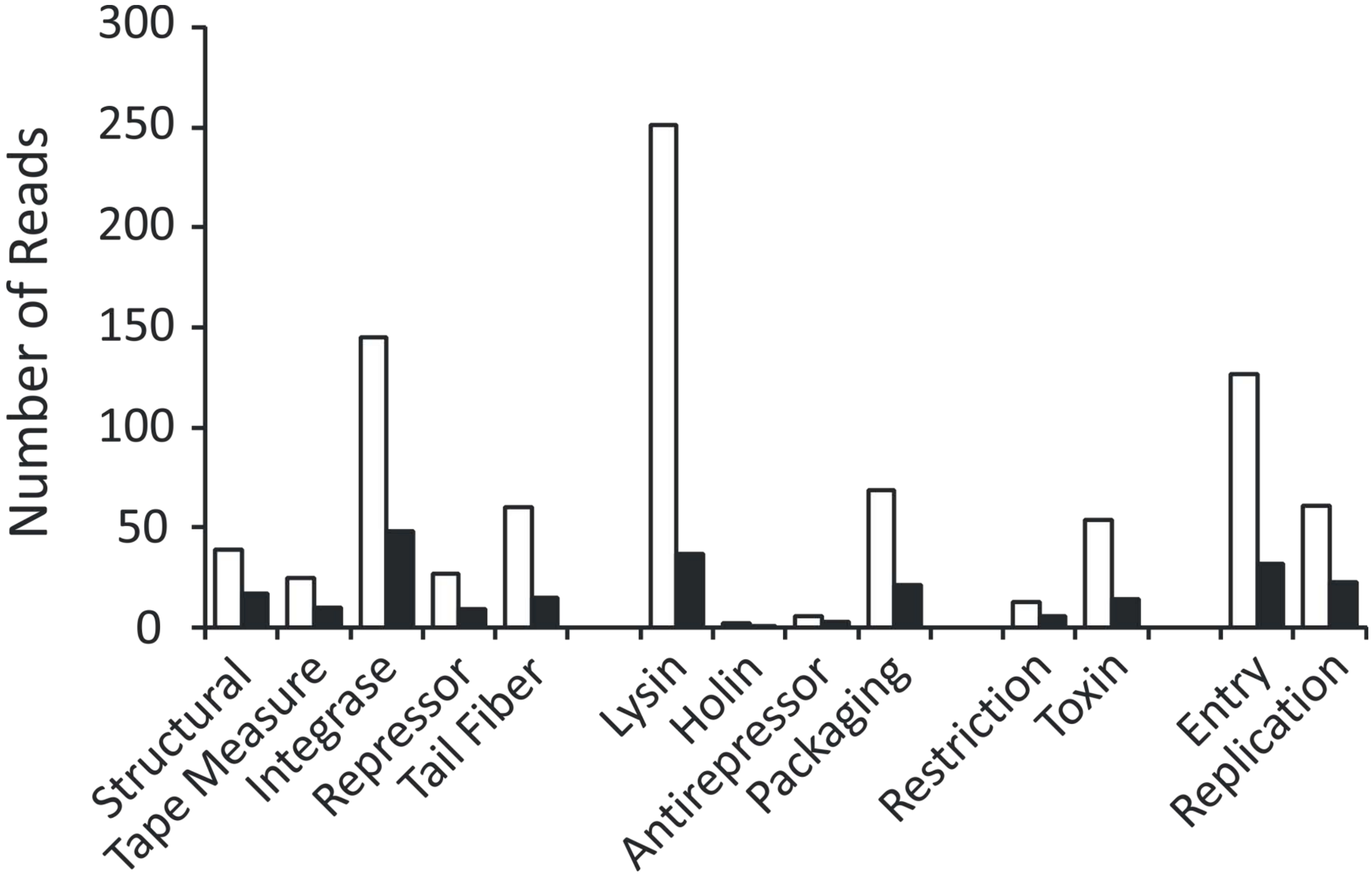

Supplemental Figure 2: Number of transcriptome reads with identifiable phage homologues in subjects with periodontal health (white bar) or disease (black bar).

Supplemental Figure 3

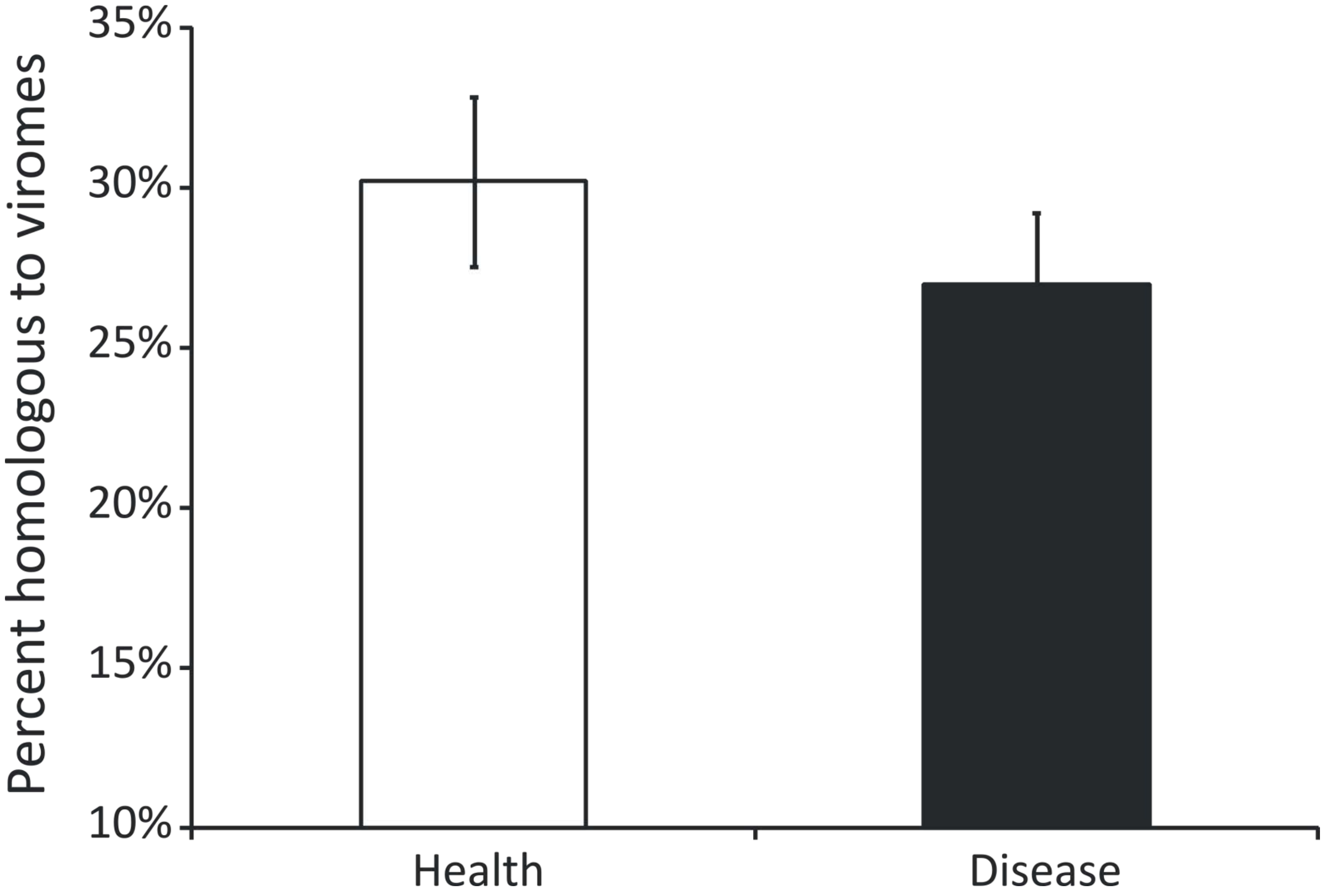

Supplemental Figure 3: Percentage (±standard deviation) of transcriptome reads from subjects with periodontal health (white bar) or disease (black bar) that were homologous to virome sequences from all subjects.
